# Temporal trends of drug requests in the Addiction Liaison Psychiatric Unit. 12 years in Hospital del Mar (Barcelona)

**DOI:** 10.1192/j.eurpsy.2023.1389

**Published:** 2023-07-19

**Authors:** M. García Jimenez, J. J. Fuentes Valenzuela, F. F. C. Fonseca Casals, M. Torrens Melich

**Affiliations:** 1 Addictions, Institut de Neuropsiquiatria i Addiccions (INAD); 2 Addictions, Institut Hospital del Mar d’Investigacions Mèdiques (IMIM); 3 Departamento de Psiquiatría y Medicina Legal, Universitat Autònoma de Barcelona, Barcelona, Spain

## Abstract

**Introduction:**

Substance Use Disorders are frequently associated to other medical problems. The temporal evolution of the main drug requests is related to drug and drug users’ facts. COVID-19 pandemic is worthy of investigation.

**Objectives:**

To analyze temporal trends in the characteristics of all medical requests to the Addiction Liaison Psychiatric Unit from January 2010 to December 2022.

**Methods:**

Study data will be obtained from all patients that were referred to the Addiction Liaision Psychiatric Unit during 12 years in Hospital del Mar (Barcelona). Demographics and clinical data (substance use, medical diagnosis, dual diagnosis) will be obtained and analyzed by semesters.

**Results:**

The results will be presented as soon as all data is obtained. We will explore COVID-19 pandemic implications.

**Conclusions:**

.

**Disclosure of Interest:**

None Declared

